# Bioinspired Protein/Peptide Loaded 3D Printed PLGA Scaffold Promotes Bone Regeneration

**DOI:** 10.3389/fbioe.2022.832727

**Published:** 2022-07-07

**Authors:** Xiaoliang Song, Xianxian Li, Fengyu Wang, Li Wang, Li Lv, Qing Xie, Xu Zhang, Xinzhong Shao

**Affiliations:** ^1^ Department of Hand Surgery, Hebei Medical University, Shijiazhuang, China; ^2^ Department of Hematological Oncology, Heji Hospital affiliated to Changzhi Medical College, Changzhi, China; ^3^ Department of Hand Surgery, The third Hospital of Hebei Medical University, Shijiazhuang, China

**Keywords:** PLGA scaffold, 3D printing, protein/peptide decoration, bio-inspired, bone defect

## Abstract

**Background:** This study was aimed to investigate the effect of three dimensional (3D)printed poly lactide-co-glycolide (PLGA) scaffolds combined with Gly-Phe-Hyp-Gly-Arg (GFOGER) and bone morphogenetic protein 9 (BMP-9) on the repair of large bone defects.

**Methods:** 3D printing method was used to produce PLGA scaffolds, and the sample was viewed by both optical microscopy and SEM, XRD analysis, water absorption and compressive strength analysis, etc. The rabbits were divided into six groups randomly and bone defect models were constructed (6 mm in diameter and 9 mm in depth): control group (*n* = 2), sham group (*n* = 4), model group (*n* = 4) and model + scaffold group (*n* = 4 rabbits for each group, 0%,2% and 4%). The rabbits were sacrificed at the 4th and 12th weeks after surgery, and the samples were collected for quantitative analysis of new bone mineral density by micro-CT, histopathological observation, immunohistochemistry and Western blot to detect the protein expression of osteoblast-related genes.

**Results:** This scaffold presented acceptable mechanical properties and slower degradation rates. After surface modification with GFOGER peptide and BMP-9, the scaffold demonstrated enhanced new bone mineral deposition and density over the course of a 12 week *in vivo* study. Histological analysis and WB confirmed that this scaffold up-regulated the expression of Runx7, OCN, COL-1 and SP7, contributing to the noted uniform trabeculae formation and new bone regeneration.

**Conclusions:** The application of this strategy in the manufacture of composite scaffolds provided extensive guidance for the application of bone tissue engineering.

## Introduction

Large bone defects generated by numerous factors, such as accidental trauma and disease, often negatively impact the normal structures and functions of the body (Liu and Lv, 2018; Chattopadhyay, 2019). The length of the bone defect exceeds 1.5 times the shaft diameter, which will lead to accelerated bone resorption and bone non-union. Therefore, the suitable materials for bone repair to treat large bone defects were still an area to be developed and researched. Since the development of bone repair materials, the functional requirements are higher and higher. The single materials promotion of osteogenic function can not meet the needs of clinical use. Composite scaffolds have good biocompatibility and bone tissue repair ability, and can really realize bone tissue reconstruction *in vivo*. It is a promising scaffold material for bone tissue engineering (Nitzsche et al., 2010; Zhou et al., 2014; Zhang et al., 2015; Kawai et al., 2017). However, the commonly used porous structure materials have weak mechanical strength, uncontrollable biodegradation rate, difficulty in revascularization, and other unfavorable factors in clinical applications (Qiu et al., 2014; Shi et al., 2015). The biodegradation rate of PDLLA is slow and the cytocompatibility of PDLLA is poor (Toda et al., 2021). However, Yi et al. (2016) incorporated PRGD and β-TCP nanoparticles into PDLLA to form composite scaffolds to improve the degradation properties, cell viability, and host tissue response of the scaffolds. Therefore, ideal biomaterials for bone-repair not only need to provide potential load-bearing support for the bone defect site, but also require a slow degradation rate to allow for new bone formation.

The preparation of tissue engineering scaffolds by three dimensional (3D) printing technology has brought hope for the repair of bone defects. 3D printing materials commonly used at present often have disadvantages such as insufficient mechanical strength, excessive impurities and degradation products that are not conducive to cell growth and differentiation (Kamali et al., 2014; Li et al., 2018). At present, the known materials of 3D printable bone scaffolds were synthetic polymers. However, these materials have their own limitations. The synthetic polymers, such as ceramics and bioactiveglass usually have low mechanical strength, poor shape retention, insufficient cell adhesion. The mechanical strength and toughness of the scaffolds decreased obviously when they were prepared as multi-pore scaffolds (Li et al., 2014). Poly lactide-*co*-glycolide (PLGA) scaffold has good biomechanical properties and controllable biodegradation rate (Yan et al., 2017). PLGA is a kind of synthetic polymer, which is a popular scaffold material in tissue process (Liu et al., 2021; Rocha et al., 2022). It has the advantages of controllable degradation rate, high porosity and low toxicity (Chia and Wu, 2014). Among the various biomaterials utilized in bone tissue engineering applications, PLGA is a superior choice when considering biocompatibility and biodegradation (Zhang et al., 2018). Moreover, the degradation rate and the mechanical properties can be tuned through control of the ratio of lactide and glycolide in the copolymer (Murphy et al., 2013). At present, PLGA is widely used in the fields of pharmacy, medical engineering materials and modern industry.

An effective modification method was to combine hydrophilic substances, such as peptides or amino acid polymers, on the surface of the scaffold. The electrostatic interaction between the positive charge on the surface and the negative charge on the cell membrane enhances the adhesion of cells to the scaffold (ZhuJiaqi et al., 2020). It has been reported that adding growth factors to the surface of polymer scaffolds can lead to cell surface interactions and stimulate growth, especially in large-scale bone defects (Gugjoo et al., 2016; Jiang and Xu, 2020). Therefore, the hydrophilic surface molecules are loaded onto the surface of the PLGA scaffold. Commonly used technologies include impregnation method (Fu et al., 2017), adsorption freeze-drying technology (Lin et al., 2015), double diffusion method (Amrita Arora et al., 2015), et al. One of the most common strategies was loading with osteoinductive molecules, aiming to enhance hydrophilicity, recruitment of stem cells and the induction of osteogenesis (Mahjoubi et al., 2014). Influenced by natural extracellular matrix (ECM) components and bone morphogenetic proteins (BMPs), a derivative peptide, Gly-Phe-Hyp-Gly-Arg (GFOGER) and BMP-9 are confirmed to stimulate osteogenesis and bone regeneration bone defect models (Bilem et al., 2016; Mansour et al., 2020). In addition, GFOGER is the amino acid sequence on the α1 chain of type I collagen (Col-I), which is the main recognition site for binding of Col-I to integrin receptor α2β1 (Emsley et al., 2000; Sipilä et al., 2014). GFOGER is type I collagen (CoL-I) α an amino acid sequence (502–507 segments) on the 1 chain is the receptor of type I collagen and integrin α2β1 main recognition sites of binding (Emsley et al., 2000; Sipilä et al., 2014). The unique triple helix sequence of GFOGER can be α2β1 is uniquely identified with BMSCs and is easy to modify materials and prepare. It can also promote the adhesion, proliferation and differentiation of osteoblasts, so as to accelerate the repair process of bone defects (Reyes and García, 2004).

Although previous studies have proved the optimal pore size and porosity of scaffolds. However, before the emergence of 3D printing technology, the scaffold materials prepared by traditional technology had some disadvantages, such as the internal pore size and porosity were difficult to be accurately controlled, and the shape could not completely match the host bone defect, so it was difficult to meet the actual needs (Ryan et al., 2006). Osteoblast cells respond to native-like scaffolds possessing a rough and highly porous environment with a high surface area for cellular migration and ECM deposition. Therefore, scaffolds used in bone tissue engineering require an adjustable structure and a shape that conforms to the bone defect site to achieve a similar effect to naturally found scaffolds (Yang et al., 2005; Sayin et al., 2014). As the pore size decreases, the overall effective surface area of the scaffold will increase. However, it should be noted that too small pore size may make it difficult for cells to adhere to the scaffold (Buj-Corral et al., 2018). Saska et al. (2018) used SLS technology to make 3D scaffolds with poly3-hydroxybutyrate as material. The results showed that the scaffolds interconnected pores can be used as an excellent tissue engineering scaffold (Saska et al., 2018). To date, many materials have been used to fabricate 3D scaffolds, such as silk fibroin, cellulose, and PLGA (Kankala et al., 2018; Chen et al., 2019; Wang et al., 2019; Zou et al., 2020). PLGA is a promising scaffold material for bone tissue engineering (Zhou et al., 2014; Zhang et al., 2015; Kawai et al., 2017). Song et al. (Song et al., 2017) mixed PLGA and collagen extracted from duck eel (DC) to prepare DC/PLGA scaffolds. *In vitro* experiments showed that the comprehensive strength of the scaffold was increased, which could promote the cell proliferation of rabbit bone marrow mesenchymal stem cells (rBMSCs) and induce them to differentiate into osteoblasts. In addition, significant up-regulation of gene expression related to osteogenic differentiation and bone regeneration was observed. Satish et al. (Ska et al., 2017) of Indian Institute of technology found that the mechanical and biological properties of HAP bioceramic composite scaffolds were significantly improved by controlling the pore distribution of scaffolds.

However, there were inconsistencies between *in vitro* cell studies and animal studies. It was suggested that the mechanism of material—mediated bone repair is not completely clear, and few bone repair materials have been approved for clinical use. Therefore, it is urgent to find an effective bone repair material. Our study revealed the effect of GFOGER and BMP-9PLGA scaffolds loaded with different concentrations on the repair of bone defects.

## Materials and Methods

### Fabrication of 3D Printed Poly Lactide-Co-glycolide Scaffolds

The molecular weight of PLGA was 60000, and the LA/GA was 75:25. The end group was carboxyl. The viscosity was 0.8 dl/g. The PLGA scaffold was fabricated *via* the melt deposition method using a three-dimensional printer (3D Bio-Architect Pro, Hangzhou Regenovo Biotechnology Co., Ltd. China). Briefly, the powder of PLGA was melted, extruded and deposited through the nozzle at 197 ± 2°C melting temperature. Finally, the porous scaffolds were solidified at room temperature. The detailed parameters, such as the shape and size were controlled by computer. Considering that the porosity, morphology and regularity of the scaffolds can be affected by the fluid flow rate due to the melting temperature, air pressure, speed and the layer thickness, the related parameters were set as follows: the inner diameter of the nozzle was 0.4 mm, the temperature of the nozzle was 197°C, the air pressure was 0.2 MPa and the air speed was 10 mm/s. The 3D print parameters aimed to fabricate uniform scaffolds in the shape of a cubic cylinder with a diameter of 6 mm and height of 9 mm.

### Physicochemical Characterization of Poly Lactide-Co-Glycolide Scaffold

The 3D printed PLGA scaffolds were analyzed by optical microcopy (CX41, OLYMPUS, Japan). The structures of the PLGA scaffold were further observed using scanning electron microscopy (SEM, FEI Quanta 250, United States). The scaffolds were immersed in normal saline at 37°C for 24 h. After removing the sample from the saline solution, the weight after water absorption was recorded using a precision balance (BSA 224S-CW Sartorius, United States). The water absorption ratio was calculated by Eq. 1.

$$\text{W }\left(\text{\%}\right)=\;\frac{\left(B-G\right)}{G}\times 100\text{\%}$$ (1)

G and B represented that mass of scaffolds before and after water absorption, respectively.

In addition, the mechanical properties of the prepared PLGA scaffold were investigated using a microcomputer-controlled electronic universal testing machine (ETM204C, Beijing Boyikang Co., Ltd, China). Press the support vertically, up and down. Printing parameters: The speed was set as 0.5 mm/min. The ambient temperature is 23.7° and the humidity is 52%.

### *In Vitro* Degradation

*In vitro* immersion testing is often used to evaluate the degree of biodegradation those scaffold materials under investigation. Briefly in this study, normal saline (0.9 NaCl saline solution) was employed as the degradation buffer. Thirty scaffolds were immersed into normal saline and incubated at 37°C. The mass ratio of scaffold to saline was 1:100. During the incubation process, three sample was weighed every week. The degradation rate is represented as the ratio of the retained mass of the scaffold in saline and its initial mass (Eq. 2).

$$\text{Degradation rate }\left(\text{\%}\right)=\;\frac{W_{i}-W_{t}}{W_{i}}\;\times 100$$ (2)

*Wi* and *Wt* were the initial mass and the retained mass of PLGA scaffold, respectively.

### Preparation of the Peptide Loaded Poly Lactide-Co-Glycolide Scaffold

GFOGER peptide is provided by Shanghai Gill Biochemical Co., LTD. The nanometer chitosan acetic acid solution (2%) was prepared before protein/peptide loading: nanometer chitosan powder was slowly added into 0.1 M acetic acid achieving a final mass volume fraction of 2%.

The 2% peptide/protein was prepared as follows: GFOGER (20 mg) and BMP-9 (20 mg) were dissolved into sterile water (500 ml) and then mixed with 5 ml of the 2% chitosan acetic acid mixture. Fifteen PLGA scaffolds were submerged in the 2% peptide/protein mixture before 500 μL of sodium glycerophosphate solution (100 mg/ml) and 500 μL of sodium bicarbonate solution (70 mg/ml) were gradually added. The mixture was incubated at 37°C for 30 min to obtain a hydrogel. Another 4 h of incubation was required to form into solid. The scaffolds were then separated and dried for 6 h. The newly loaded scaffold materials were kept at −20°C.

The final amount of GFOGER peptide and BMP-9 used in the 4% protein/peptide group were 40 mg. In addition, the scaffolds without peptide/protein incorporation but with chitosan hydrogel decoration were identified as the 0% control group.

### *In Vitro* Peptide Release

The *in vitro* release assay was achieved according to the procedures previously reported (Yang et al., 2005). In brief, the peptide-decorated scaffold was incubated in 10 ml centrifugation tubes supplemented with 4 ml of PBS at 37°C. At predetermined time points (1, 2, 4, 8, 12, 24, 48, 72, 96, 120 and 144 h), 50 μL of the liquid was harvested and an equal volume of fresh PBS was added. The precise amounts of the released peptides were detected using UV spectrum (absorbance at 280 nm) and the cumulative release rate was calculated to evaluate the *in vitro* release behavior.

### Animal Study

22 New Zealand rabbits were procured from Wangdu Tonghui Beering Co., Ltd. (Shijiazhuang, China). The room in which the animals were housed had filtered air at the rate of 10–20 air changes per hour. The temperature was maintained at 16–26°C with a relative humidity of 40%–70%. Temperature and humidity were continuously monitored and daily minimums and maximums recorded. Illumination was fluorescent light for 12-hour light (8:00–20:00) and 12-hour dark. Animals had libitum access to rabbit food and tap water. All animal studies were administrated by The Third Hospital of Hebei Medical University (Shijiazhuang, China).

### Establishment of Bone Defect Model and Materials Implantation

The rabbit bone defect model was established according to the reported studies (Zheng et al., 2014). Briefly, all 20 rabbits were anesthetized by pentobarbital sodium (2 ml mg/kg weight) through intravenous injection. Twenty of the 22 rabbits were randomly selected to undergo surgery, 16 of which were made into bone defect models, and 4 were not made into bone defect models, but were simply opened and sutured. The posterior legs were shaved, disinfected by 75% ethanol three times. A skin incision was made over the lateral femur-tibia joint, the femoral distal condyle was exposed after the muscle and fascia tissues were peeled away. A bone defect area (6 mm in diameter and 9 mm in depth) in the dual lateral femoral condyle was created. Following the defect preparation, the surgical area was thoroughly rinsed with physiological saline to wash out any bone fragments. The scaffolds were implanted (*n* = 4 rabbits for each group, 0, 2% and 4%), *n* = 4 rabbits were included in a model group (no scaffolds implantation), *n* = 4 rabbits were included in a sham group (bone defects are not constructed, only simple incision and suture), and *n* = 2 rabbits in the control group. Three days after surgery, each rabbit was injected with penicillin (800,000 U/d) for infection prevention. Animal movement was not restricted and the rabbits were fed following the same method as was used prior to surgery.

Two rabbits from control group were anesthetized and sacrificed on day 7. The femurs identified in the same positional space as those in the experimental groups were isolated and kept for further characterizations. The rabbits in the other experimental groups were sacrificed on weeks 4 and 12. They were investigated with gross anatomical observations. The local bone callus formation was noted after sacrifice and the original incision and the bilateral femoral tissues (2 cm) were harvested after removal of soft tissues for further analysis.

### Micro CT Analysis

Micro CT was used for the quantitative analysis of new bone formation and the extent of degradation of the bone-repair scaffolds. The 3D structures were reconstructed using 1,536 scanned slices. The threshold values ranging from 1,000 to 3,000 related to new bone and those values exceeding 3,000 was considered artificial bone. For each specimen, a cubic cylinder with a diameter of 2 mm and a height of 2 mm was taken from the three vertices of the middle triangular cross-section perpendicular to the vertical axis of the scaffold, and the quantitative value of new bone mineral density was measured. As control, five cubic cylinders with a diameter of 2 mm and a height of 2 mm, were randomly selected from the cross-section. Each cubic cylinders contained complete artificial bone. Finally, artificial bone density and bone mass were measured and the three dimensional images were reconstructed.

### Histological Analysis

Histopathology (hematoxylin-eosin staining and Masson Trichrome staining) was applied to investigate the formation of new bone in the bone defect. Additionally, the expression of osteogenic factors including runt-related transcription factor 2 (Runx2) (Ab-AF5186,Affinity), osteocalcin (OCN) (OM266707, OmnimAbs)and Collagen I antibody (bs-10423R,Bioss) were analyzed through immunohistochemistry.

### Western Blot

The bone tissue was ground into powder by liquid nitrogen, and then ground again after adding lysate. The tissue homogenate was sucked into an EP tube, lysed on ice for 30 min, and centrifuged at 12000 rpm for 10 min. By absorbing supernatant, total protein can be obtained. Protein denaturation, sample loading, sodium dodecyl benzene sulfonate gel electrophoresis (SDS-PAGE) for 2 h. It was incubated with primary antibody (BS-1110R, Bioss, 1/1000; AF5186, Affinity, 1/1000) overnight, 4°C. The second antibody (ZB-2301, Zhongshan Jinqiao, 1/2000) was incubated in the solution at room temperature for 2 h. ECL exposure solution was added to the membrane and exposed in the gel imaging system.

QuantityOne software (BioRad) was used to analyze the expression of the different bands of protein.

### Statistical Analysis

All the data were presented as mean ± standard deviation (SD). The data were plotted using Graphpad Prism software and the statistical analysis was applied using SPSS 19. Significant difference between groups were analyzed by two-way ANOVA (variance is homogeneous) or Rank Sum Test (variance is not homogeneous). And unless specified, *p* < 0.05 was considered as a significant difference.

## Results

### Characteristic of Scaffold

**FIGURE 1:**
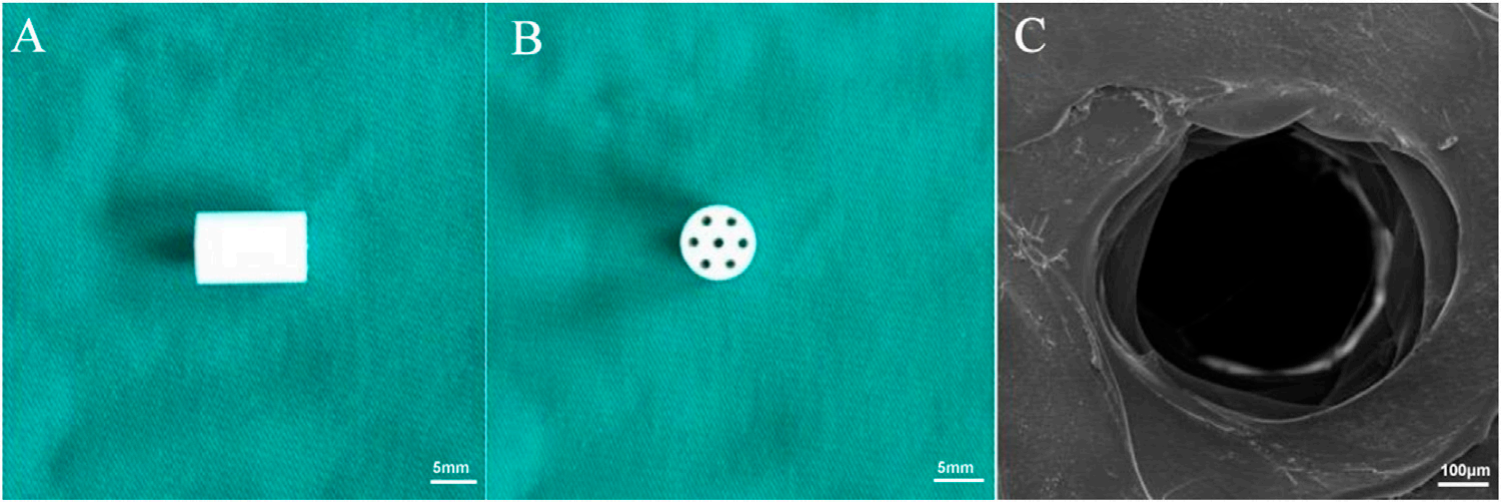
Morphology of the 3D printed scaffolds. **(A)** The side view and **(B)** top view of the prepared scaffolds. **(C)** SEM images of PLGA scaffolds.

The cylindrical scaffolds fabricated by 3D printing technology displayed regular morphology (Figures 1A,B). The macroporous morphology with a resulting pore size of 400 ± 35 μm. Further confirming the porous structure of the 3D printed scaffolds, the SEM image exhibits a defined and full depth pore in the compact polymers (Figure 1C). Basic condition of the scaffold (Table 1).

**TABLE 1:** Basic condition of the scaffold.

Scaffold types (%)	Repeat	Scaffold size
0	4	Diameter 6 mm, height 9 mm
2	4	
4	4	

**FIGURE 2:**
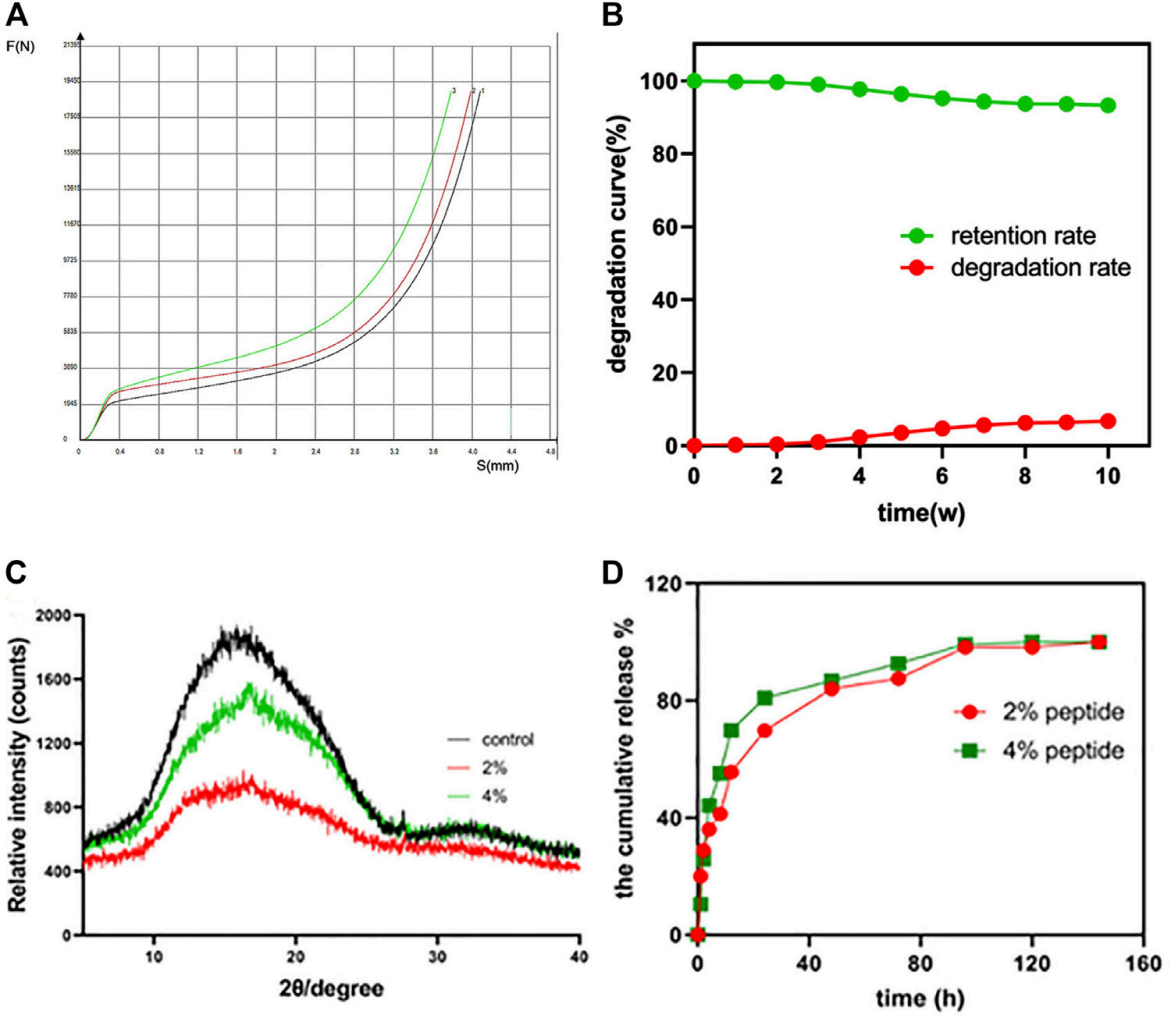
**(A)** The compressive strength (force-displacement curve) of the PLGA scaffold (*n* = 3). **(B)** The *in vitro* degradation behavior of the PLGA scaffold. **(C)** XRD patterns of the raw PLGA scaffold and the peptide-loaded scaffolds; **(D)** the *in vitro* peptide release profile of the 2% and 4% peptide-loaded PLGA scaffolds.

As shown in Figure 2A, the X-axis represents the distance of the displacement (mm). And the Y axis represents the amount of the pressure (N). It was a compression test. These results were similar to those of PLDLA/PCL-T Scaffold and rapid prototyped PCL scaffolds (Esposito et al., 2013). Scaffolds for use in bone tissue engineering applications attempt to achieve 19450 ± 50 N (mean value ± standard deviation) in order to provide structural support. When the maximum stress range was exceeded, the bracket will be irrevocably deformed, indicating that the bracket has certain compressive resistance. The average dry weight was 0.32 ± 0.0.004 g, and the average wet weight was 0.34 ± 0.007. The average absorption rate of the 3D PLGA scaffolds at 24 h was approximately 7% ± 0.81%. It was indicated that the 3D printed PLGA scaffold allows for water absorption to a certain degree, indicating a suitable environment for peptide incorporation and cell adhesion.

During the study, due to the mechanical stability, the degradation rate was noted to gradually increase, ultimately reaching 7% at 10 weeks (Figure 2B). The XRD patterns of PLGA scaffolds, before and after peptide loading, are shown in Figure 2C. There are no other peaks in the control group. The halo-shaped peaks characteristic of PLGA indicate that the stent is in an amorphous state, similar to the previously confirmed PLGA raw material. Moreover, after loading of the GFOGER peptide and BMP-9 protein, no changes were noted in the XRD scan, further confirming the introduction of biological factors do not induce structural transformation.

The cumulative release of the GFOGER peptide and BMP-9 from the PLGA scaffolds was illustrated in Figure 2D. Both the 2% and 4% peptide loaded scaffolds displayed similar release behavior, resulting in an early burst release then reaching sustained release over time. It was note that the release rate of the 2% peptide groups was slower than that of the 4% peptide groups. It was indicated that the greater loading efficiency can achieve effective drug concentration in a short time (48 h). There was no significant difference in the final release, and both reached 100%. These data suggest that the 3D printed PLGA scaffold serves as an effective scaffold for loading and releasing peptide/protein drugs for tissue engineering applications.

### New Bone Formation

**FIGURE 3:**
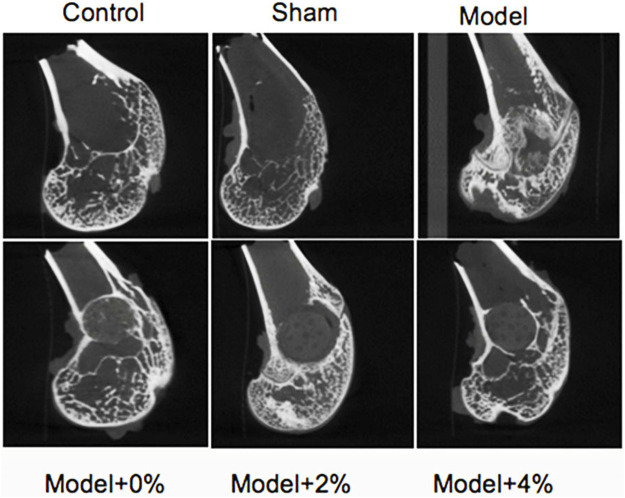
New bone density after 12 weeks as analyzed by micro CT.

Four weeks after surgery, the sham group exhibited normal bone structure compared with the control groups. While obvious bone defects were observed in the bone defect model groups, the implanted scaffolds (0%, 2% and 4%) were connected closely to the surrounding bone tissue without significant biodegradation. At 12 weeks after surgery, all of the scaffolds studied had degraded partially and uncovered the merger of new bone and surrounding bone tissues. Micro-CT was employed to explore the impact of these loaded scaffolds on new bone formation in the bone defect models (Figure 3). The new bone densitiesare listed in Table 2 at 12 weeks after surgery. Compared with the sham group, the new bone density of the model group was lower, yet with incorporation of peptide/protein loaded molecules the bone density increased. Specifically, the scaffold containing 4% GFOGER peptide and BMP-9 presented the best bone-repair effect, highlighting the synergistic effect of mechanical support from the PLGA scaffold and the pharmacoinvasive strategy, GFOGER peptide and BMP-9 intervention.

**TABLE 2:** New bone density after 12 weeks as analyzed by micro CT.

Groups	New bone density (mg/cc)
Control group	4067.29 ± 604.94
Sham group	4498.77 ± 1297.48
Model group	4133.74 ± 951.90
Model + 0% group	4917.57 ± 1181.95
Model + 2% group	5035.35 ± 1347.36
Model + 4% group	5503.94 ± 1412.33

Note: Pairwise comparisons between groups showed no difference.

### Histological Analysis

**FIGURE 4:**
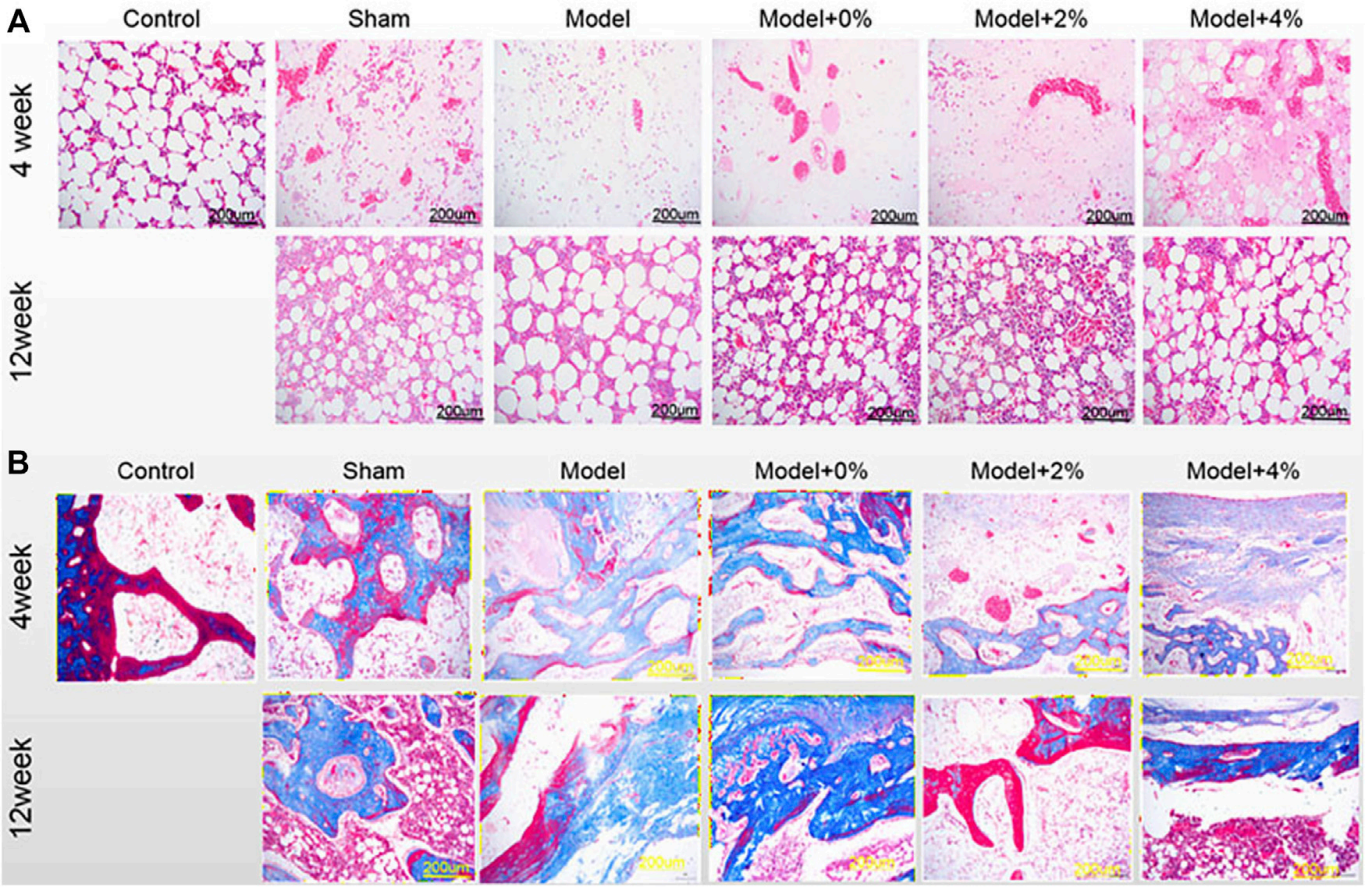
**(A)** H&E staining and **(B)** Masson-trichrome staining of the bone tissues at 4 and 12 weeks post-surgery. (200×).

The extent of bone regeneration at 4 and 12 weeks post-surgery was further evaluated by H&E staining and Masson Trichrome staining. At 4 weeks post-surgery, the control group exhibited normal morphology and structure, while the defect site in the model group was completely filled with fibrillar connective tissue (Figure 4A). Those defect sites with implanted PLGA scaffolds (0%, 2% and 4%), resulted in an increased population of chondrocytes and osteocytes. In all the PLGA model groups, the formation of new bone trabeculae appeared and there were fibrous tissues surrounding the edges of the bone grains. The scaffold containing 4% chitosan (with GFOGER peptide and BMP-9) displayed the greatest new bone integration with existing host tissue. At 12 weeks post-surgery, abundant fibrocytes and little bone tissue aggregation were noted in the model group, while the neatly arranged trabeculae appeared in the area surrounding the bone defects as seen in all the PLGA model scaffolds. In addition, the medullary cavity and the new bone formation were repaired as expected.

Another method used to evaluate the degree of bone maturation was the Masson-Trichrome stain (Figure 4B). The limited or reduced degree of red stain indicates new bone tissue, while the deeper red stain indicates mature bone tissue In the control group, Masson staining was mainly red, that is, mainly mature bone. At 4 weeks post-surgery, the red stained area decreased in the model group and model +0% group, while the green stained area increased, which means that new bone tissue was the main part. But in the model 2% and 4% group, the red and the green stained region coexisted, and the green stained area was reduced compared with the model group. Moreover, at 12 weeks with the increase of red staining area, green staining area decreased significantly, indicating that the mature bone tissue was gradually increased.

### Immumohistochemical Staining of Osteogenic Molecules

**FIGURE 5:**
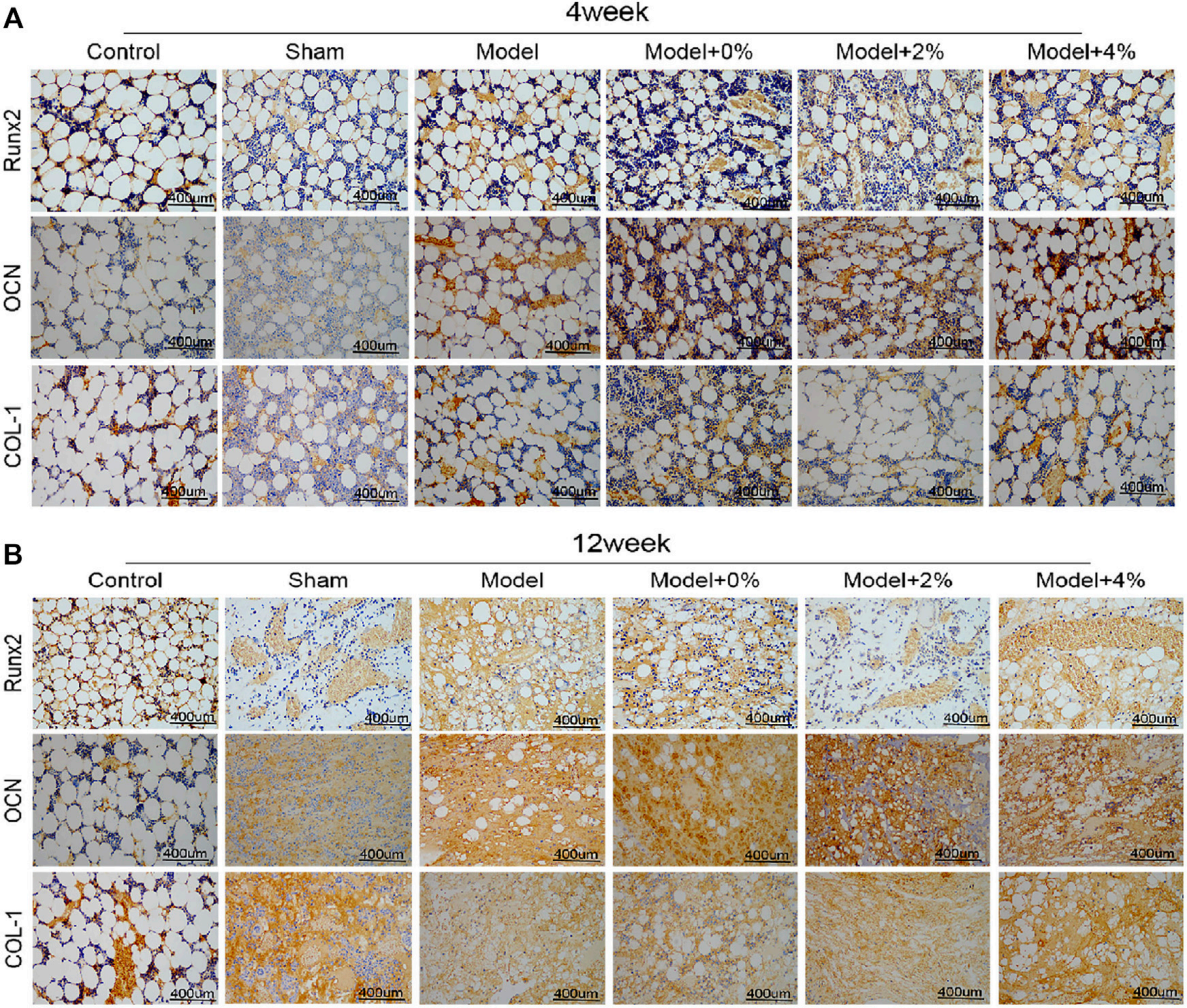
The immumohistochemical staining for detecting the expression of Runx2, OCN and COL-1 at 4 **(A)** and 12 **(B)** weeks (400 ×).

The immumohistochemical staining was used to evaluate the effects of the implanted scaffolds on cellular up-regulation of bone-related factors (Runx2, OCN and COL-1) in the bone-defect models. As shown in Figure 5, the expression levels of Runx2, OCN and COL-1 as seen in the model group were higher than those of the control group. In addition, treatment of the bone defect site with the 3D printed PLGA scaffold models demonstrated expression levels were higher than those observed in the model group alone. Additionally, the expression of Runx2, OCN and COL-1 at 12 weeks post-surgery was notably higher than that observed at 4 weeks. All the obtained data indicated that the implanted 3D printed bone-repair scaffolds used in this study could promote new bone formation.

### Western Blot Analysis

**FIGURE 6:**
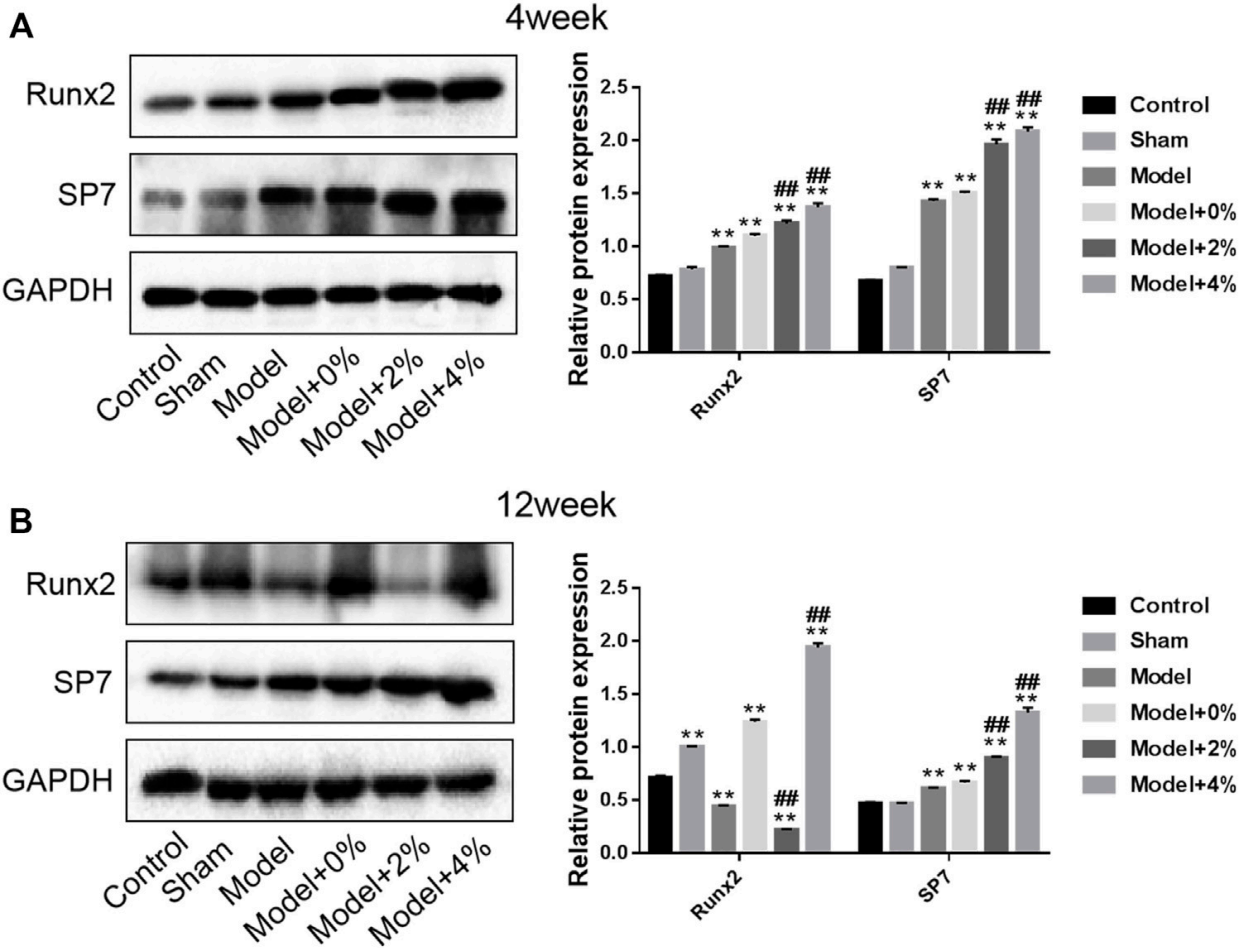
Western blot analysis for determination of the expression of Runx2 and SP7 protein at 4 and 12 weeks. Compared with the Control group, **p < 0.01; compared with the Model group, ^##^ *p* < 0.01.

In order to further investigate the effect of the implanted PLGA scaffold on bone formation in the bone defect models, the expression of Runx2 and SP7 protein using Western blot was employed. At 4 weeks post-surgery, the expression of these two factors in the model group was significantly higher than those in the control group (*p* < 0.01). And this tendency was enhanced after treatment with the 3D printed PLGA scaffolds (0%, 2% and 4%) (Figure 6). Furthermore, at 12 weeks post-surgery, the natural bodily response results in an up-regulation of SP7 and a down regulation of Runx2. However, treatment with the 4% chitosan hydrogel-incorporated PLGA scaffold overcame the native response to further up-regulate both Runx2 and SP7 proteins.

## Discussion

An ideal implanted scaffold for tissue engineering applications consists of biodegradable materials and-bioactive factors, which is capable of being degraded slowly during the regeneration process to allow for the innervation and development of blood vessels and bone tissues (Gil-Albarova et al., 2012). Synthetic polymers typically have low mechanical strength, poor shape retention, poor cell adhesion, and may release potentially toxic degradation products in the body (Brody, 2015). Taking into account the inherent shortcomings of currently used materials, the creation of composite scaffolds fabricated with particular polymeric ratios to allow for fine-tuned degradation. These composite materials can not only retain the special properties of each components, but also enhance the composite material with newly endowed synergistic properties not seen in the single component (Tatara and Mikos, 2016). In the meanwhile, utilizing precise 3D printing technology presented an improved alternative. The porous scaffold material prepared by 3D printing technology can accurately control the size and porosity of the inner pore of the scaffold by designing the generation path in advance to ensure the growth of bone tissue and blood vessel regeneration (Xu et al., 2015). Therefore, composite materials are much more likely to achieve the requirements of those scaffolds required for bone tissue engineering applications. At present, there are different categories Type materials, composite materials with additional bioactive factors and nano-composite materials appeal to many more researchers.

PLGA was chosen as the raw material to fabricate scaffolds using 3D printing technology for the following reasons: 1) it is a clinical synthetic materials approved by the FDA due to its non-toxic behavior (Zhao et al., 2016); 2) it is easy for processing and surface modification with good mechanical strength and capacity in controlled release (Gobbi et al., 2009); 3) the biodegradation rate can be tuned through altering the ratio of PLA and PGA in this copolymer; 4) it is compatible for cell survival and proliferation and it can induce tissue repair (Thi Hiep et al., 2017). Therefore, we chose PLGA as the stent because of its obvious advantages. The mechanical strength, water absorption and peptide release rate of PLGA stent were tested. Tissue repair was induced by adding bioactive molecules GFOGER and BMP-9. According to the theory of Holmes (Holmes, 1979), the average pore size of bones of human beings is approximately 223 μm and the optimal pore size of the implanted materials should be controlled in the range of 200–400 μm. When the pore size is larger than 300 μm, it proves beneficial for cell migration, proliferation and blood vessel growth. In addition, Tsuaga *et. al* confirmed that the ideal pore size of ceramics that support ectopic osteogenesis is 300–400 μm (Tsuruga et al., 1997). Therefore, the pore size of the 3D printed PLGA scaffold in this study was tuned to achieve 400 μm (Figure 1), which is in accordance with the aforementioned studies. The inherent hydrophobicity of PLGA is not conductive to cell seeding. Yet, the higher water absorption suggested that this scaffold has the possibility to accept peptide/protein loading and sustained release. Thus improving the biocompatibility and osteoinductive capacity of the scaffold can be achieved through functionalizing the materials with biomacromolecules.

BMP-9 is endowed with the best ability to induce osteogenesis and it cannot be suppressed by BMP-3 (Dumanian et al., 2017). BMP-9 has also been confirmed to induce other cells into osteocyte differentiation, such as osteoblasts, pre-osteoblasts and stem cells (Wang et al., 2014). Another biological factor in this study was the GFOGER peptide, which can promote osteogenic differentiation, bone regeneration, bone integrity, and accelerate osteogenesis in the bone defect model (Mhanna et al., 2014). Therefore, when implanted into the bone defect model, the PLGA scaffolds with or without peptide/protein loading demonstrated improved bone regeneration behavior. Among them, the PLGA with 4% GFOGER peptide and BMP-9 had the best advantage in promoting bone regeneration. Furthermore, the biodegradable by-products did not negatively impact the bone-repair process. Among various osteogenic factors, Runx 2 is expressed in osteoblast progenitor cells. The highest expression of Runx2 occurs before osteoblasts reach full maturation and then it gradually reduces after maturation (Oliveira et al., 2019). OCN is not only a marker of osteoblastic function and bone mineralization, but also a specific index for evaluating the bone formation and bone conversion rate (Yu et al., 2020). COL-1 is the most abundant collagen in the human body and the only type of collagen found in mineralized bone, thus its metabolites of synthesis and decomposition indirectly reflect the status of bone transformation (Wallace et al., 2010; Oliveira et al., 2019). In this study, PLGA with 4% GFOGER peptide and BMP-9 up-regulated Runx2, OCN, COL-1, and SP7, which indicated that this composite scaffold was an ideal candidate in bone tissue engineering applications. All in all, in our experiment, different concentrations of polypeptides were used to detect different effects on bone defect repair. As a result, the uniform trabeculae formation and new bone regeneration are most significantly improved after a 12-week bone defect model implantation study.

Although this study highlights the synergistic and complementary effects of GFOGER and BMP-9 as well as the advantages of scaffold surface functionalization and verifies the positive role of composite scaffolds in bone defect repair. But the structural design and mechanical properties of this research was not in-depth enough. This study only conducted a descriptive analysis and comparison of the results, not a systematic statistical comparison. Later, we will conduct more detailed experiments to enrich our experimental data.

## Conclusion

In general, 3D printed PLGA scaffold (with 4% GFOGER and BMP-9) is capable of up-regulating the expression of Runx2, OCN, COL-1 and SP7 to enhance bone regeneration in the bone defect model. This strategy of integrating biological components into 3D printing scaffolds has great scientific potential in the application of bone tissue engineering. Moreover, with the development of 3D printing technology and biomedical engineering, more functional scaffolds can be applied in bone tissue engineering.

## Data Availability

The datasets used and/or analyzed during the current study are available from the corresponding author upon reasonable request.
